# A Proteogenomic Approach to Unveiling the Complex Biology of the Microbiome

**DOI:** 10.3390/ijms251910467

**Published:** 2024-09-28

**Authors:** Luciana Alexandra Pavelescu, Monica Profir, Robert Mihai Enache, Oana Alexandra Roşu, Sanda Maria Creţoiu, Bogdan Severus Gaspar

**Affiliations:** 1 Department of Morphological Sciences, Cell and Molecular Biology and Histology, Carol Davila University of Medicine and Pharmacy, 050474 Bucharest, Romania; luciana.pavelescu@umfcd.ro (L.A.P.); monica.profir@rez.umfcd.ro (M.P.); oana-alexandra.rosu@rez.umfcd.ro (O.A.R.); 2 Department of Oncology, Elias University Emergency Hospital, 011461 Bucharest, Romania; 3 Department of Radiology and Medical Imaging, Fundeni Clinical Institute, 022328 Bucharest, Romania; robert-mihai.enache@rez.umfcd.ro; 4 Department of Surgery, Carol Davila University of Medicine and Pharmacy, 050474 Bucharest, Romania; bogdan.gaspar@umfcd.ro; 5 Surgery Clinic, Bucharest Emergency Clinical Hospital, 014461 Bucharest, Romania

**Keywords:** genomics, proteomics, metabolomics, microbiota

## Abstract

The complex biology of the microbiome was elucidated once the genomics era began. The proteogenomic approach analyzes and integrates genetic makeup (genomics) and microbial communities′ expressed proteins (proteomics). Therefore, researchers gained insights into gene expression, protein functions, and metabolic pathways, understanding microbial dynamics and behavior, interactions with host cells, and responses to environmental stimuli. In this context, our work aims to bring together data regarding the application of genomics, proteomics, and bioinformatics in microbiome research and to provide new perspectives for applying microbiota modulation in clinical practice with maximum efficiency. This review also synthesizes data from the literature, shedding light on the potential biomarkers and therapeutic targets for various diseases influenced by the microbiome.

## 1. Introduction

The gut microbiota in humans is a complex ecosystem primarily composed of four major bacterial phyla: *Bacteroides*, *Firmicutes*, *Proteobacteria*, and *Actinobacteria*. These microorganisms play a crucial role in various physiological processes that are essential for maintaining human health. They contribute to protecting against pathogen invasion, processing and extracting nutrients from the diet, balancing energy metabolism, and regulating immune responses. The balance and composition of these gut microbes can significantly influence health outcomes, including susceptibility to diseases, metabolic disorders, and immune-related conditions [1,2].

**Figure 1:**
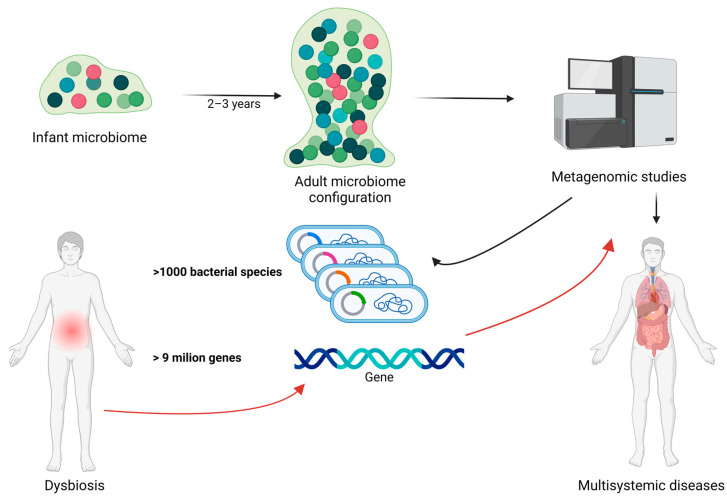
The relationship between gut microbiota, dysbiosis, bacterial species/genes, and metagenomic studies in human health. The gut microbiota undergoes significant development during the first 2–3 years of life, eventually establishing the mature adult microbiota. Metagenomic studies can identify the genes and bacterial composition of the gut microbiota, potentially playing a crucial role in early detection of genes involved in dysbiosis and related diseases. Black arrow represent the normal evolution and characterization of the intestinal microbiome during life. Red arrows represent the importance of metagenomic studies in dysbiotic circumstances. Created with BioRender.com (accessed on 3 September 2024).

The evolution of the gut microbiota begins immediately after birth and undergoes continuous and significant changes, particularly during the first 2–3 years of life. During this period, the microbial composition rapidly diversifies until a stable adult microbiota is established. Metagenomic studies have shown that the human gut microbiota is highly complex, comprising over 1000 bacterial species and more than 9 million genes, with substantial variation in composition and diversity among individuals. Disruptions to this delicate balance, known as dysbiosis, can lead to significant changes in the gut′s metaproteome, potentially contributing to various health issues including obesity and other nutrition-related diseases [1,2]. In Figure 1, we illustrate the relationship between gut microbiota, dysbiosis, bacterial species/genes, and metagenomic studies in human health.

**Figure 2:**
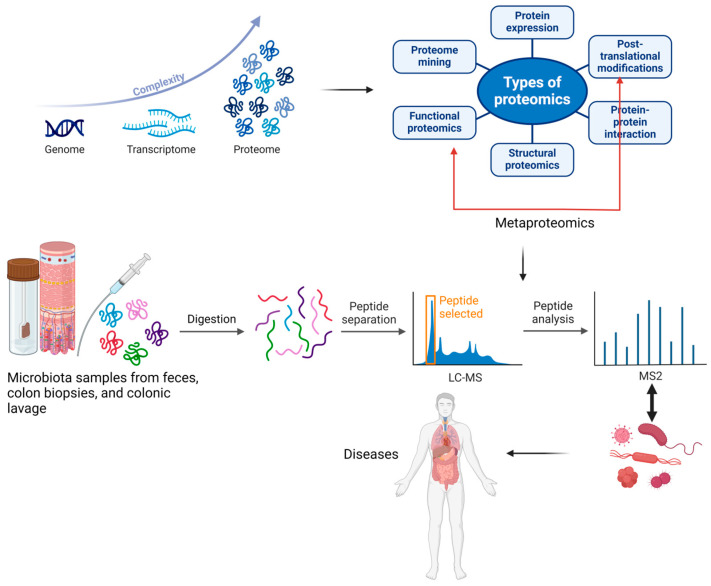
The development of the proteome and metaproteome and their use in analyzing proteins and associated microbial species from feces, colon biopsies, or colonic lavage samples, along with their applications in discovering various diseases. The blue arrow indicates the complexity of the improved analytical techniques, which led to the discovery of a multitude of peptides following the post-translational process. The red arrow indicates the importance of functional proteomics in identifying the roles proteins play in carrying out cellular processes at the molecular level. Created with BioRender.com (accessed on 3 September 2024).

Metaproteomics, a branch of proteomics, was defined for the first time by Wilmes and Bond as “the large-scale characterization of the entire protein complement of environmental microbiota at a given point in time”. While proteomics examines all proteins within a single species, metaproteomics allows for investigating proteins from multiple organisms. Metaproteomics enables the characterization of proteins and their associated microbial species by identifying translational and post-translational modifications that may lead to various diseases. Research indicates that post-translational modifications such as deamination, acetylation, methylation, hydroxylation, oxidation, nitrosylation, and phosphorylation allow bacteria to adapt to changing environmental conditions [1,3]. In Figure 2, we have illustrated the evolution and utility of metaproteomics in analyzing proteins and associated microbial species, highlighting its applications in discovering various diseases.

Various bioinformatics databases (such as NCBI-nr, X! Tandem, and GigaScience) are currently employed for protein and peptide identification, including high-sequence coverage databases (which are time-consuming and less sensitive in peptide identification) and synthetic databases (which enhance specificity by incorporating known gut microbial genome sequences, but lack a comprehensive map). Additionally, emerging approaches such as multi-step database searches and the integration of metagenomic data into metaproteome databases are being utilized [1,4].

Although metaproteomics is a powerful tool for investigating the protein content of samples, it comes with certain limitations. It requires specialized algorithms and software, increased financial investment, and larger storage capacities for taxonomic and functional annotation. Additionally, constructing databases for protein identification and managing redundant protein grouping can be complex challenges [5].

Looking ahead, future studies should explore the potential of metaproteomics for applications in process control for technical purposes or routine diagnostics using fecal samples. As the number of applications and sample volumes grows, there will be a need for software with greater memory capacity, faster analysis times, and user-friendly interfaces suitable for both medical and non-medical personnel. Moreover, new and enhanced bioinformatic strategies will be essential to improve the identification of spectra [5]. The main purpose of this review is to integrate and summarize the new omics technologies and diverse challenges regarding their applications in microbiome research, a field that has expanded rapidly in the last decade. We also focused on the significance of new biomarkers and therapeutic targets for different multisystemic diseases that can occur or be amplified by intestinal dysbiosis. Proteogenomic technologies will be enhanced in their significance and interpretation with the aid of artificial intelligence and machine learning, which are rapidly expanding.

## 2. Applications of Genomics, Proteomics, and Bioinformatics in Microbiome Research

Recent studies using metagenomic data from fecal samples of two premature infants have revealed significant shifts in bacterial populations and notable differences between their microbiotas. While metagenomic analysis highlighted community variations and potential gene proteins, metaproteomics provided deeper insights into real-time functional signatures, such as metabolic activity and host-microbe interactions during gut colonization [6,7]. The application of high-performance mass spectrometry in these studies has enhanced proteome coverage in complex samples, and when combined with matched metagenomes, it has led to more accurate and confident protein identification [2,6,7]. This combination of metagenomics and metaproteomics has shown remarkable success in analyzing complex microbiota samples, offering a more comprehensive understanding of gut microbial dynamics [2].

The most common approach for analyzing proteins in the gut microbiota is mass spectrometry-based shotgun metaproteomics, utilizing samples from feces, colon biopsies, and colonic lavage [1]. This technique surpasses traditional shotgun proteomics by employing diverse methods, including bead-beating, ultrasonication, freeze-thawing, heating, and detergents, to identify and characterize a broad spectrum of Gram-negative and Gram-positive bacteria [8]. Metaproteomics surpasses metagenomics by evaluating the functional activity of a microbe rather than merely analyzing its genomic potential [9].

Matrix-assisted laser desorption/ionization time-of-flight mass spectrometry (MALDI-TOF) is a technology adopted in microbiology laboratories. Its primary advantage lies in its rapid acquisition of bacterial spectra, enabling the differentiation of microorganisms across various genera and species. This capability was demonstrated in a study by Seng et al., which successfully identified 84% of bacteria at the species level and 11% at the genus level. Looking ahead, MALDI-TOF holds potential for identifying antibiotic susceptibility and subtyping bacterial species, aiding in the detection of bacterial strains or clones linked to infection outbreaks [1,10].

Recently, metaproteomics has been integrated with metabolomics and 16S rRNA gene sequencing to investigate and characterize shifts in the abundance of specific bacteria, proteins, and metabolites within the human gut microbiota in response to dietary resistant starch [1,11]. In a study by Maier et al., a customized sequence database revealed that the bacterial families *Bacteroidaceae*, *Ruminococcaceae*, *Lachnospiraceae*, and *Prevotellaceae* exhibited the most significant protein identifications [4]. Additionally, the study observed an increased *Firmicutes*-to-*Bacteroidetes* ratio and a rise in proteins involved in lipid metabolism associated with a high-resistant starch diet [11]. Another study by Kolmeder et al., which compared obese and non-obese patients using an in-house composite database, found that 73% of identified peptides could be classified down to the phylum level or lower. A combination of metaproteomic and phylogenetic data revealed a reduced abundance but heightened activity of *Bacteroidetes* in obese patients compared to non-obese individuals [12]. Recent studies have emphasized the importance of integrating metagenomics, metaproteomics, metatranscriptomics, and meta-metabolomics to understand microbiome functions comprehensively [8].

Metaproteomics can characterize various diseases influenced by dysbiosis, such as inflammatory bowel diseases (including Crohn′s disease, ulcerative colitis, and irritable bowel syndrome), gastric cancer, and type 1 diabetes [1]. In studies analyzing fecal samples from patients with Crohn′s disease, a reduction in bacterial diversity in the ileum was observed, along with a depletion of proteins from the *Firmicutes* phylum and metabolic pathways involved in hydrocarbon degradation, butyrate, and other short-chain fatty acid production [13]. Another study on Crohn′s disease found significant differences between metaproteomic and metagenomic datasets despite consistent taxonomic associations at the genus level [13,14]. A metaproteomic study examining fecal samples from patients with inflammatory bowel disease, irritable bowel syndrome, colon adenoma, and gastric carcinoma identified metaproteins primarily assigned to the human host (75%) and microbial communities (25%) [15]. In another metaproteomic study focused on the fecal microbiome of patients with newly diagnosed type 1 diabetes, researchers detected a decrease in exocrine pancreas proteins and an increase in immune-modulatory proteins, such as fibrillin-1 and galectin-3 [16]. The study also found a correlation between the bacterial species *Alistipes* and *F. prausnitzii* with a cluster of exocrine pancreas-produced proteins, mucosal epithelial barrier proteins (MUC2 and FCGBP), and the adhesion molecule CEACAM5 [16].

## 3. Defining the Normal Intestinal Microbiota—Possibilities, Challenges and Pitfalls

In recent years, research has increasingly concentrated on creating comprehensive and standardized databases to better understand the global diversity of normal gut microbiota. Despite variations due to human genetics, environmental factors, dietary habits, and the growing availability of sequencing data, it remains unclear whether all species and genes within the gut microbiome have been identified and whether there are common or rare elements yet to be discovered [17].

The most commonly used methods for analyzing fecal samples include targeted 16S rRNA gene pyrosequencing and metagenomic shotgun sequencing, though the latter involves higher costs and more complex data analysis. Additionally, culture-based or single-cell approaches are employed. Metagenomic assembly of short sequencing reads offers functional insights and provides genomic information on environmental microbes in a more convenient and unbiased manner compared to other methods. Today, metatranscriptomics and metaproteomics are also increasingly utilized [17].

With the help of 16S rRNA sequencing, studies have shown that Bacteroidetes and Firmicutes represent the majority of the distal gut microbiota [18]. In a study by Qin et al., an Illumina-based metagenomic sequencing method was employed to characterize the gut microbiota from fecal samples of 124 European adults, including those who were healthy, obese, or had inflammatory bowel disease. Their gene catalog comprised 3.3 million microbial genes, representing the most known human gut bacterial genes, with 99.96% assigned to Bacteria and Archaea. These included members of the *Bacteroidetes*, *Dorea*, *Eubacterium*, *Ruminococcus*, *Streptococci*, and *Lactobacilli* groups, as well as *Bifidobacteria* and *Proteobacteria*. Table 1 presents the bacterial species most frequently identified in the intestinal microbiota [18].

The authors highlighted that patients with inflammatory bowel disease in their cohort exhibited lower bacterial diversity, with 25% fewer genes compared to healthy individuals. They also observed that individual variability in gut microbial species significantly impacted the identification of a common core microbiota. Despite identifying 57 species present in 90% of individuals, with *Bacteroidetes* and *Firmicutes* as the dominant genera, interindividual variability remained considerable. The study emphasized the importance of sampling depth, noting that a 2- to 3-fold increase in sequencing depth resulted in a 25% rise in the number of species detected. They estimated that no more than 15% of bacterial genes were likely missed. Furthermore, the authors acknowledged that future studies might complement their findings, as the gut microbiota is continually evolving, and sequencing methods continue to advance [18].

Li et al. successfully created a universal gene catalog by combining databases from Europe, America, and Asia, revealing population-specific characteristics of gut microbiota. This integrated gene catalog, covering strains with diverse abundance, occurrence frequencies, and transit durations in the human gut, enhances the mapping of sequencing reads from the cohorts used for its construction, accounting for 94.5% of the gene content in the sampled gut microbiome. As a result, it stands as a valuable resource for future gut microbiota studies. The authors ensured high-quality sequencing reads by applying stricter quality controls with the FASTX Toolkit, revising assembly in the MOCAT pipeline, using an improved assembler, SOAPdenovo 1.06, and employing a standardized, ultrafast clustering algorithm (CD-HIT) to merge gene catalogs, along with more specific gene calling via MetaGeneMark version 3.25 [17,18,20,21,22].

Their study found no significant differences compared to other studies regarding bacterial species with increased frequency in the gut microbiota, except for variations primarily influenced by diet-related processes (such as energy, carbohydrate, amino acid, cofactor, and vitamin metabolism), xenobiotic-associated functions, and host genetics. A reduction in *Enterococcus* levels was generally observed across most fecal samples, although occasional higher occurrences were noted in Chinese and European populations. The Chinese population exhibited significantly lower α-diversity in both genes and genera. Compared to the Chinese population, which showed an abundance of *Proteobacteria*, the gut microbiota of European populations, particularly Danish individuals, had a higher presence of *Firmicutes*, including *Oenococcus* and other lactic acid bacteria. Notably, *Oenococcus*, a genus typically not considered commensal in the gut microbiota, had an increased frequency of 13.5% in their cohort [17]. In addition to bacteria, the study also identified more eukaryotes than other studies, although pathways such as proteoglycan biosynthesis, glycosphingolipid biosynthesis, and diterpenoid biosynthesis were absent [17]. Table 1 presents the bacterial species most frequently identified in the intestinal microbiota.

In the study conducted by Li et al., the importance of closely monitoring cohort behaviors, particularly their exposure to antibiotics, was emphasized. The researchers identified increased penicillin resistance genes in Danish populations and multidrug resistance genes in Chinese populations despite participants reporting no recent antibiotic use. They also highlighted various factors that can influence efforts to define normal gut microbiota. These factors include microbes present in food and drinks, quantitative data on microbial intake and excretion, the half-life of specific strains in the gut, the presence of particularly abundant strains that may indicate a deviation from health or reflect specific environmental conditions, and the fact that colonoscopy can detect more microbes than fecal sampling alone [17].

In a study by Schloissnig et al., the authors analyzed stool samples from 207 European and North American individuals using metagenomic shotgun data. They identified 66 dominant species, which accounted for 99% of the mapped reads. A notable finding from the study is that host conditions, such as genetic differences, immune tolerance, and diet, have less influence on species evolution compared to factors common to the human population, like gut physiology, anaerobic conditions, and pH [20]. Schloissnig et al. also highlighted that each individual has a unique metagenomic variation profile, and healthy individuals tend to retain specific strains over extended periods. The authors acknowledged that their study spanned one year, which may not be sufficient for a comprehensive analysis, and they emphasized the need for larger datasetss with standardized sampling and sequencing protocols for more detailed insights [19].

Zhang et al. introduced a novel metaproteomic method called MetaProteome Identification and Quantification (MetaPro-IQ) in their study, designed to bypass the need for metagenomic sequencing when analyzing the gut microbiota of humans and mice [4,17,23]. This method utilized the human/mouse gut microbial gene catalog. At the outset, the authors emphasized two significant limitations: the vast number of proteins from various studies and the extensive range of gene catalog databases, which constrained the sensitivity of database searches. To address this, they implemented an iterative database search strategy that reduced the database size and consequently enhanced search sensitivity. In their human study, mucosal-luminal interface samples were collected during endoscopies from eight children, identifying 67,186 distinct peptides corresponding to 19,011 protein groups. The authors proposed that their approach achieved superior identification and quantification of microbial proteins compared to metagenomic databases. Additionally, their findings revealed similar phyla abundance in the microbiota as other metaproteomic studies, with *Firmicutes*, *Bacteroidetes*, *Verrucomicrobia*, *Proteobacteria*, and *Actinobacteria* as the dominant phyla, along with an elevated *Firmicutes*-to-*Bacteroidetes* ratio [4].

In a study by Kolmeder et al., the researchers examined the fecal metaproteome of three healthy individuals throughout 6 to 12 months using a novel high-throughput technique. This method combined denaturing polyacrylamide gel electrophoresis with liquid chromatography–tandem mass spectrometry alongside metagenome and single-genome sequence data. They identified at least 1000 peptides per sample and observed significant temporal stability in the metaproteome, which aligned with the microbial composition. This finding suggests a strong association between the composition and function of the intestinal microbiota. In their cohort, glutamate dehydrogenase emerged as the most abundant protein across various bacterial families, including *Lachnospiraceae*, *Bacteroidaceae*, *Ruminococcaceae*, and *Bifidobacteriaceae*. Beyond its primary metabolic role in the gut microbiota—linking the nitrogen and carbon cycles by incorporating ammonia into 2-ketoglutarate—glutamate dehydrogenase also functions as an electron sink, facilitating the conversion of pyruvate and ammonia into alanine. This process requires the activity of aminotransferases, which were also identified in the metaproteome. Other abundant proteins in their cohort included xylose isomerase and glutamine synthetase. The dominant bacteria identified were *Firmicutes* (particularly *Clostridium* cluster XIVa and *Clostridium* cluster IV), *Bacteroidetes*, and *Actinobacteria* [24].

In a review by Lee et al., the authors assessed numerous studies that utilized metaproteomic analysis to examine gut microbiota composition and function. They noted that studying the gut microbiota is challenging and fraught with obstacles [25]. For instance, various studies demonstrated that storage temperatures significantly influence the microbial profile, with frozen intact fecal material proving more stable [26,27,28,29]. Additionally, a notable bias in metaproteomic studies arises from sample preparation; while centrifugation can increase protein identification, it also results in considerable protein loss due to the non-specific removal of microbial cells. Free-treated stools are considered to provide a more accurate representation of microbial proteins [25].

In a study by Xiong et al., a double filtering separation step was employed, effectively depleting human proteins and selectively enriching microbial proteins, which enhanced proteome coverage by facilitating the identification of low-abundance proteins [2]. The method of protein extraction is also crucial; bead beating, for example, has been found to be particularly efficient for Gram-positive bacteria [30,31]. Secretome studies, which use fecal samples to identify secreted proteins, often require extensive clean-up procedures, leading to protein loss. The choice of software for metaproteomic data analysis is also very important [2].

One of the most common challenges in metaproteomic studies is the highly diverse microbial community and the vast protein dynamic range, which can be partly addressed by using capillary and microchip methods, albeit with increased time and costs. Quantitative metaproteomic analysis is still evolving to identify better microbial genes involved in metabolic functions. The most commonly used quantitative techniques include protein-based stable isotope probing, label-free quantification, and metabolic labeling for improved peptide quantification [2].

## 4. Current Methods for Studying the Intestinal Microbiome

Studying the intestinal microbiome has become a crucial area of research due to its significant impact on human health, disease, and overall well-being. The intestinal microbiome refers to the complex community of microorganisms living in the gastrointestinal tract, including bacteria, viruses, fungi, and archaea [32]. Understanding this ecosystem is vital for unraveling its role in digestion, metabolism, immune function, and disease processes such as obesity, diabetes, and inflammatory bowel disease (IBD) [33,34,35,36].

In recent years, advances in technology and methodologies have greatly enhanced our ability to study the intestinal microbiome. Thus, various technologies, including DNA and RNA sequencing techniques and metabolomic and metaproteomic techniques, are now being used for the study of the microbiome [37].

Culture-based methods using standard bacterial culture techniques, which involve growing microorganisms in controlled laboratory conditions using selective media, were initially used for assessing gut microbiome composition. However, the fact that a large number of intestinal bacteria are obligate anaerobes and are unable to survive the processes of collecting, transport, and storage represents a major disadvantage. Moreover, bacteria differ in their tendency to grow in different cultures, leading to a cultivation bias in favor of aerobic bacteria [38].

The need for more precise methods of analysis led to the development of DNA sequencing techniques, one of the most powerful tools in microbiome research. One of these methods is 16S rRNA gene sequencing, a molecular assessment technique that identifies bacterial species based on their 16S ribosomal RNA (16S rRNA) genes. 16S ribosomal RNA (rRNA) gene sequencing is a widely used method for identifying and classifying bacteria within the microbiome. The 16S rRNA gene is highly conserved among bacteria, making it a reliable marker for phylogenetic studies [39]. Multi-parallel sequencing techniques involve several steps, including sample collection, DNA extraction, PCR amplification, sequencing, and data analysis [40]. 16S rRNA Gene Sequencing techniques have the advantage of allowing the analysis of multiple samples simultaneously and of being relatively inexpensive compared to whole-genome sequencing [39]. Whole-genome shotgun (WGS) sequencing is also a molecular sequencing technique that involves sequencing the entire genetic material in a sample, providing a comprehensive view of the microbiome, including bacteria, archaea, viruses, and fungi [41]. The process requires four steps: sample collection (fecal or intestinal tissue), DNA extraction, sequencing the whole genome via high-throughput sequencing platforms, and, finally, data analysis [42]. In contrast to 16S rRNA sequencing, shotgun metagenomics sequences the entire genome of all organisms present and allows the characterization of the genetic and genomic diversity along with the functional potential of the microbial domains and offers the possibility to assign taxonomy at the species and strain levels [41].

Another sequencing tool is meta-transcriptomics, which involves the extraction and sequencing of RNA instead of DNA. While DNA sequencing techniques offer the possibility to determine the functional capacity of the genomic material of the bacteria found in a microbial community, they lack the capacity to determine if these genes are expressed or not. The gene expression profile can be assessed by studying the RNA. Furthermore, meta-transcriptomics can capture real-time responses to environmental changes, making it a great tool with the potential to uncover biological information that other sequencing tools cannot reveal [43].

Metaproteomics involves the study of proteins expressed by the microbiome, offering insights into microbial function and interactions with the host. This method provides information on protein expression and function and allows the investigation of host-microbe interactions. However, it is less sensitive than DNA/RNA-based methods and requires advanced expertise in proteomics and bioinformatics [44].

The functional potential of the microbiota can also be studied through metabolomics, which focuses on the study of metabolites produced by the microbiome [45].

Gut microbiome functions are being unraveled thanks to the abundance of data being produced by the rapidly expanding fields within genomic studies, such as metagenomics, and non-genomic analyses, such as proteomics and metabolomics, together with bioinformatics tools [46]. Given that the microbiome is also transcriptionally regulated, understanding the role of the gut microbiota requires the application of metatranscriptomics and metaproteomics [43]. Table 2 presents the most important methods and their roles in studying the intestinal microbiome.

## 5. Proteomics

Proteomics represents the study of the expressed proteins from a specific genome [47]. Unlike genomic or transcriptomic analyses, which focus on DNA or RNA, proteomics provides direct information on the proteins that are being actively expressed by microbes in a given environment, thus reflecting the real-time functional state of the microbiome [48]. The rapid growth of this field has transformed it into a powerful tool for discovering biomarkers and altered protein levels related to specific diseases, leading to a better understanding of many disease mechanisms [49].

The first mass spectrometry (MS)-based proteomic analysis from fecal samples from healthy donors was conducted in 2009 by Verbekmoes et al. with an aim to better understand the complex interactions between microorganisms in different microbial communities [50]. Since 2009, the field of metaproteomics has further developed. The methodology of metaproteomics involves protein extraction, trypsin proteolysis, and the resulting peptides analysis via MS, which can be combined with liquid chromatography. The data are further analyzed, matching the recorded MS against a protein sequence database [51]. To identify bacterial species from protein samples analyzed by MS, researchers rely on gene catalog databases generated from human and animal models. These databases generate high protein coverage and offer the possibility of comparing results. The key bacterial databases are UniProt, NCBI RefSeq, Ensembl and UniRef [52]. For the gut microbiome study, there are several biological sources available, including mucous samples, biopsy specimens, and fecal samples. Fecal samples have the major advantage of being easily obtainable through non-invasive techniques [53]. Fecal samples are complex mixtures containing bacteria and human cells along with food content and substances from different sources such as drugs or inorganic material, but studies have shown that mass spectrometry can differentiate between these sources [54]. Microbial cells from fecal samples must first be enriched via differential centrifugation to enable high-sensitivity metaproteomic analysis. The quality of metaproteomic studies is highly dependent on the efficacy of protein extraction and several methods such as differential centrifugation, bead-beating, ultrasonication, and freeze-thawing have been developed for that purpose [55].

The identification and quantification of proteins from mixed microbial communities can be difficult, especially when dealing with poorly characterized or unculturable species [48]. The application of gut metaproteomics would not have been possible without high-performance workflows used for the identification and quantification of intestinal microbial proteins, such as MetaPro-IQ [4]. Due to the high workload and low sensitivity associated with analyzing metaproteomic databases, software tools such as MPA Portable 2.0 and MetaPro-IQ have been introduced to simplify the search and increase the accuracy of protein identification [55].

After sample collection and proteome analysis, bioinformatics is used to discover potential biomarkers or signaling pathways. Proteomic data are complex and require sophisticated bioinformatics tools for analysis. One approach is de novo identifying proteins from complex heterogeneous mixtures, which seems like the logical solution because many metaproteomes have not been characterized until now. However, this method can lead to many false identifications [56]. A target-decoy database search strategy is the preferred technique for accurately identifying proteins and peptides, saving researchers from the need to distinguish between correct and incorrect peptide identification, which is required with most sequence search engines [57].

MaxQuant is the most frequently used quantitative proteomics software package, offering quantitative and normalized protein abundance information and offering the possibility for comparison across samples [58].

The identification of gut-related proteins/ peptides offers the possibility of identifying biomarkers for gastrointestinal diseases such as inflammatory bowel diseases and colorectal cancer [59,60,61]. Numerous studies demonstrating the key role the gut microbiota plays in the development of several diseases have led to the conclusion that microbiota-related proteins should also be analyzed along with host proteins [33,35,36,62,63,64,65].

Inflammatory bowel diseases (IBDs) are a group of chronic diseases of the gut represented by ulcerative colitis (UC) and Crohn disease (CD), characterized by the inflammation of the intestinal mucosa [66]. The pathophysiology of IBDs is not completely understood, and with the help of metaproteomic studies, researchers have tried to unveil its secrets. By combining shotgun metagenomic and metaproteomic techniques, Erickson et al. observed that stool samples from CD patients differ from the stool samples coming from healthy patients, as observed through a number of several proteins and pathways [13]. The metaproteomic analysis of mucosal samples from patients suffering from IBD and healthy patients revealed that proteins in the intestinal mucosa clustered into modules with different functions and cellular origins. Specific modules have been associated with UC, CD, and healthy states [67]. In addition, Zhang et al. have demonstrated that microbial proteins associated with stress responses are upregulated in patients with IBD compared to controls and that these alterations are induced by microbial alterations [68]. Interestingly, fecal metaproteomic analysis of IBD patients has demonstrated that obesity could improve the microbial diversity of UC patients and showed that obese UC patients presented higher abundance of some bacteria such as *Parabacteroides distasonis*, *Alistipes indistinctus* and *Ruminococcus bromii* [69]. One recent study has identified four biomarkers for IBD that outperform fecal calprotectin [70]. MMP-8, TIMP-2, fecal fibrinogen, and fecal PGRP-S have the potential of being used for the screening of IBD and in pediatric populations and for prognostic purposes [71].

Proteomics have an especially important role in the study of cancer, helping fill in the gap between oncogenesis and the alterations of the gut microbiome. In 2022 in a study combining microbiome, metabolome, and proteome analysis, Qian et al. demonstrated that the gut microbiome, along with its specific metabolites and differentially expressed proteins, play a key role in the pathogenesis of non-small cell lung cancer (NSCLC). The proteome analysis highlighted eight differentially expressed proteins involved in the IL-8 and NF-κB pathways and identified Lipopolysaccharide binding protein (LBP), C-reaction protein (CRP) and CD14 as potential biomarkers for NSCLC [70].

The alterations of the intestinal microbiome in CRC (colorectal cancer) patients have been intensively studied. However, the mechanisms by which these alterations lead to carcinogenesis are not yet entirely understood [37]. Integrating metaproteomic studies is taking us one step further towards understanding this process better. One study observed quantitative variations of microbial proteins in patients with CRC compared to healthy controls. These proteins mainly relate to oxidative stress and iron intake/transport. Characterizing the gut microbiome of CRC patients and healthy patients through quantitative metaproteomics has identified 341 proteins strongly associated with oxidative stress and iron intake and transport that significantly differ in abundance between the two groups [72].

One of the most important roles of proteomics is the identification of new potential biomarkers. A recent study identified 16 proteins that can distinguish between CRC patients, patients with adenomas, and healthy patients. MMP9, haptoglobin, myeloperoxidase, fibrinogen, and adiponectin are the proteins that presented the highest ability to distinguish between these groups for Caucasian patients. These proteins are potential stool CRC biomarkers and have outperformed stool hemoglobin [73]. Further studies are needed to assess the clinical applicability of these proteins.

A study integrating proteomic stool analysis of patients with CRC has also identified two proteins, SIAE and CDHR5, which play a role in maintaining intestinal epithelial function, as potential CRC biomarkers due to their capacity to distinguish between CRC and controls [74].

## 6. Metabolomics

Metabolomics studies small molecules typically involved in a biological process as a substrate or product within a mass range of 50 to 1500 Da (daltons) [75].

Several fields use metabolomics, such as illness diagnostics, studies of food and nutrition, and research and development in the pharmaceutical industry [76].

Based on certain estimates, a solitary plant possesses a wide range of metabolites, numbering from 7000 to 15,000 [77,78]. It has been estimated that all plant species contain approximately 200,000 metabolites. It is estimated that approximately 3000 endogenous or common metabolites are found in humans. These estimates may not be completely accurate, since it is challenging to detect compounds present in very small amounts [79,80].

Since they capture only a single moment under specific and specialized conditions, the analytical methods currently used in metabolomics research are constrained by their inherent limitations. We may see, in a manner comparable to that of a microbiologist, that many processes are continually taking place inside the cells. The quantities of metabolites produced as a consequence of these interactions are characterized by a great degree of dynamism and may rapidly fluctuate across consecutive periods [81,82].

At any given moment, the metabolome represents the complete assortment of metabolites found within a cell, tissue, or biological sample being studied. Small molecules are constantly consumed, produced, and broken down. They also interact with other molecules in various biological systems and the surrounding environment. The metabolome is inherently highly dynamic [83,84].

When it comes down to it, a metabolomics experiment can identify and analyze any tiny molecules, especially those with molecular weights lower than 1500 Da [84,85]. However, the experiment′s perspective is limited because of the constraints imposed by the extraction, ionization, and detection of molecules [83,86].

Considering its great sensitivity and its capacity to detect and quantify a large variety of chemicals in complex biological samples, MS (mass spectrometry) is often used for metabolomics studies [85].

Mass spectrometry, sometimes known as MS for short, is a method of analysis that identifies minute components as ions and then utilizes that identification to determine the number of molecules present in the sample. As far as omics analysis is concerned, it is a helpful tool. GC-MS (Gas chromatography–mass spectrometry), an abbreviation for gas chromatography-mass spectrometry, is a method that may be used to ascertain whether a material is volatile concerning its properties [87].

Then, derivatization techniques might be applied to make the chemical volatile. Numerous applications have shown that the GC-MS metabolomics technology efficiently analyzes primary metabolism. This has been demonstrated via several studies. The examination performed using this method is comprehensive and consists of several procedures [88,89]. Derivatization is the process that is required to transform metabolite extracts into derivatives that are both volatile and thermally stable [90]. This is the major difference between GC-MS metabolomics and other analytical platforms for metabolomics, such as liquid chromatography-MS [91].

The field of metabolomics is largely concerned with determining the concentrations of naturally occurring metabolites that are present in biological fluids and/or tissues. Research in the field of metabolomics has the remarkable capability of rapidly identifying any unfavorable alterations that are taking place in the human body. It is commonly known among researchers that differential metabolites are generally recognized as trustworthy markers of physiological activity. This is because differential metabolites are non-invasive and possess a high level of sensitivity [92]

These methods are restricted in their capacity to adequately define the characteristics of organic acids and lipids, which are both significant kinds of metabolites. On the other hand, they provide more accurate results when attempting to characterize different metabolites, such as microbial metabolites. Since this is the case, more development is necessary to achieve a much wider metabolic range [92].

## 7. Perspectives

Numerous studies have demonstrated that alterations in the microbiota, particularly dysbiosis, are linked to various host diseases. These changes can be detected using both traditional methods and cutting-edge technologies, such as advanced DNA sequencing and proteogenomic approaches, which hold significant promise for the future of personalized medicine [93]. Fecal microbiota transplantation is a therapy where microbiome analysis proves especially valuable in treating *Clostridium difficile* infection [94]. This approach is also relevant for other conditions, including inflammatory bowel diseases, obesity, metabolic syndrome, type 1 diabetes, cardiovascular disease, autoimmune disorders, certain cancers, and central nervous system disorders [95,96,97]. Other studies have highlighted the potential of probiotics and prebiotics as therapeutic approaches for chronic diseases [97].

In recurrent *Clostridium difficile* infection cases, patients typically exhibit reduced levels of *Bacteroidetes* and *Firmicutes* and decreased species richness. However, metaproteomic and metagenomic studies have demonstrated that the variability of the recipient′s microbiota is restored with normal commensal bacteria approximately two weeks after fecal transplantation, with *Bacteroidetes* becoming the dominant group [98,99].

Chen et al. conducted a study analyzing the fecal microbiota of a single patient over 14 months, both in a healthy state and during two viral infections. Using an integrative personal omics profile, which combined genomic, transcriptomic, proteomic, metabolomic, and autoantibody approaches, they identified several medical risks: modest risk for coronary artery disease and significant risk for basal cell carcinoma, hypertriglyceridemia, and type 2 diabetes. The study also revealed dynamic changes in molecular components and biological pathways associated with the individual′s health and disease conditions. They identified several genes associated with diseases and potential therapeutic options. For instance, while the TERT gene has been linked to acquired aplastic anemia in other studies, Chen et al.′s analysis of telomere length revealed little to no decrease in telomere length and only a modest increase in the number of cells with short telomeres compared to age-matched controls [100,101]. Additionally, during a respiratory syncytial virus infection, they observed a viral inflammation response that led to secondary aberrant glucose metabolism, increasing the risk of type 2 diabetes. In their study, the genes GCKR, KCNJ11, and TCF7 were associated with hypertriglyceridemia and diabetes, while the LPIN1 and SLC22A1 genes were linked to a favorable response to antidiabetic drugs like rosiglitazone and metformin [100].

Recent studies have emphasized the significant role of the microbiota in influencing the pharmacokinetics and pharmacodynamics of drugs, which can lead to varying treatment responses among patients. For instance, the therapeutic efficacy of an orally administered drug can be influenced by gut microbiota-mediated absorption. Additionally, numerous studies have demonstrated that antibiotic treatments can alter gut microbiota, impacting other drugs′ pharmacokinetics. This highlights the critical importance of microbiota modulation, alongside genetic background, in developing personalized medicine [93,97,102,103].

In a study by Bebek et al., the authors evaluated the correlation between hygiene and head and neck squamous cell carcinoma and observed increased methylation of the MDR1, IL8, RARB, and TGFBR2 genes in tumor samples. They found that MDR1 is associated with a specific microbiome profile and highlighted its potential role in bacterial-driven carcinogenesis. The study suggests that P-glycoprotein, produced by MDR1, may recognize cancer therapy drugs and impact their efficacy [104,105]. Additionally, butyrate-producing bacteria, which provide energy to the gut epithelium, regulate host–cell responses, inhibit histone deacetylase, and potentially influence gene expression and cellular processes in the gastrointestinal epithelium and beyond, represent another important aspect of the transcriptomic profile that warrants further investigation [97]. This raises the question of whether minocycline, an antibiotic effectively used in depression, might exert its effects by modulating gut butyrate production [106].

Over the past decade, extensive studies have demonstrated that proteomics can advance significantly in terms of speed, sensitivity, reproducibility, automation, and throughput. Future research should focus on developing new proteomic strategies to identify prognostic biomarkers for those requiring treatment and predictive biomarkers to evaluate the effectiveness of these therapies, particularly in patients with cancer or rare diseases, thereby advancing personalized medicine. Recent oncology studies have revealed that a proteogenomic approach can uncover new targeted therapies, although further optimization is needed to achieve maximum efficacy. As we advance, new technologies, such as enhanced antibody validation to increase specificity and the adoption of resources like the Human Protein Atlas and Antibodypedia, are expected to surpass current technologies like microarrays, aptamer technologies, and proximity assays [107,108,109].

The significance of the plasma proteome has grown in recent years, becoming a crucial tool in the biomedical and pharmaceutical fields and enhancing the validity of drug targets. In a review by Suhre et al., the authors emphasized the potential for future studies to explore human health at the molecular level by integrating proteomics with molecular readouts such as DNA methylation, metabolomics, and glycomics. This approach could deepen our understanding of diseases and foster the development of new translational strategies through the identification of targets and biomarkers [110,111].

**Figure 3:**
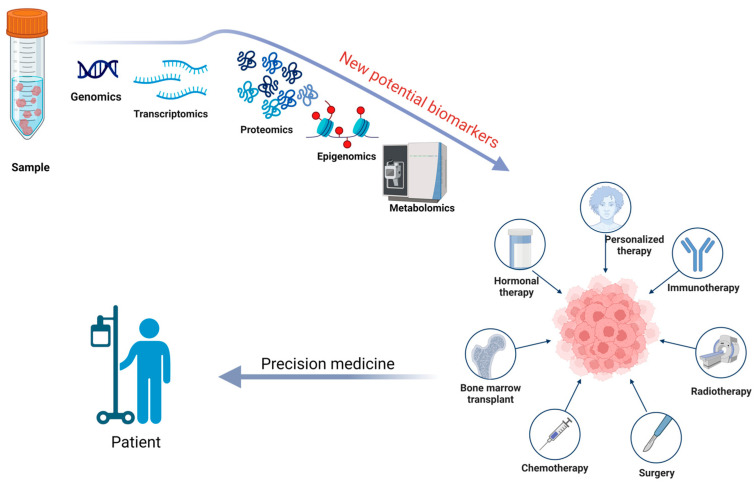
A proteogenomic approach to sample analysis can help identify new potential biomarkers for prevention, diagnosis, prognosis, and surveillance, while also determining the most effective therapeutic plan for a patient through the lens of precision medicine. Created with BioRender.com (accessed on 22 September 2024).

Additionally, future research should focus on analyzing fecal proteomics through a proteogenomics approach to understand the complex biology of the microbiome better. The prediction, diagnosis, and treatment of diseases, along with understanding the onset, progression, and prevalence of disease states, can be enhanced by combining detailed molecular analysis of samples with genomic information [100]. This will be essential for discovering and validating new potential biomarkers, particularly for oncological treatments, such as in colorectal cancer [93,107]. In Figure 3, we have illustrated the utility of a proteogenomic approach in personalize medicine.

## 8. Conclusions

In this review, we summarized key data and challenges (such as new equipment, public health systems, databases, effective approaches, interoperability between clinical and laboratory technologies, advanced tools, healthcare issues, and omics data) and discussed future perspectives for the application of genomics, proteomics, and bioinformatics in microbiome research, which is driving advancements in precision medicine. Given the numerous challenges, recent studies have emphasized the role of artificial intelligence (AI) in addressing these issues, particularly in genomics, by improving the quality, accuracy, and speed of predictions across the genome analysis pipeline. AI is also making strides in MS-based proteomics, enabling the prediction of nearly all analytical properties measured by this inherently multi-dimensional technology, leading to significant progress in precision medicine. As AI continues to improve, it is expected to enhance the accuracy of peptide and protein quantification, paving the way for novel approaches to disease diagnosis and management [112,113].

## Figures and Tables

**Table 1:** Some studies define normal gut microbiota.

Study	Normal Gut Microbiota	Total Number of Bacterial Species Identified
Qin J et al. [18]	*Blautia hansenii*, *Clostridium scindens*, *Enterococcus faecalis*, *Clostridium asparagiforme*, *Bacteroides fragilis*, *Bacteroides intestinalis*, *Ruminococcus gnavus*, *Anaerotruncus colihominis*, *Bacteroides pectinophilus*, *Clostridium nexile*, *Clostridium* sp., *Parabacteroides johnsonii*, *Bacteroides finegoldii*, *Butyrivibrio crossotus*, *Bacteroides eggerthii*, *Coprococcus eutactus*, *Bacteroides stercoris*, *Holdemania filiformis*, *Clostridium leptum*, *Streptococcus thermophilus*, *Bacteroides capillosus*, *Subdoligranulum variabile*, *Ruminococcus obeum*, *Bacteroides dorei*, *Eubacterium ventriosum*, *Bacteroides* sp., *Coprococcus comes*, *Bacteriodes xylanisolvens*, *Eubacterium rectale*, *Bacteroides ovatus*, *Parabacteroides distasonis*, *Eubacterium siraeum*, *Roseburia intestinalis*, *Bacteroides vulgatus*, *Dorea formicigenerans*, *Collinsella aerofaciens*, *Ruminococcus lactaris*, *Faecalibacterium prausnitzii*, *Ruminococcus* sp., *Unknown* sp., *Ruminococcus torques*, *Eubacterium hallii*, *Bacteroides thetaiotaomicron*, *Bacteroides caccae*, *Ruminococcus bromii*, *Dorea longicatena*, *Parabacteroides merdae*, *Alistipes putredinis*, *Bacteroides uniformis*.	57 species
Li et al. [17]	*Eubacterium*, *Alistipes*, *Ruminococcus*, *Clostridium*, *Parabacteroides*, *Odoribacter*, *Dorea*, *Bifidobacterium*, *Butyrivibrio*, *Sutterella*, *Akkermansia*, *Oscillibacter*, *Dialister*, *Lactobacillus*, *Victivallis*, *Oribacterium*, *Mobiluncus*, *Lactococcus*, *Thermoanaerobacterium*, *Abiotrophia*, *Turicibacter Bacillus*, *Peptoniphilus*, *Acetivibrio*, *Oenococcus*, *Cellulosilyticum*, *Alkaliphilus*, *Treponema*, *Ethanoligenens*, *Slackia*, *Lawsonia*, *Pelotomaculum*, *Epulopiscium*, *Caulobacter*, *Brachyspira*, *Methanobrevibacter*, *Escherichia*, *Klebsiella*, *Streptococcus*, *Shigella*, *Enterococcus*, *Veillonella*, *Campylobacter*, *Enterobacter*, *Citrobacter*, *Anaerostipes*, *Salmonella*, *Fusobacterium*, *Granulicatella*, *Actinomyces*.	50 species
Schloissnig et al. [19]	*Collinsella stercoris*, *Enterococcus faecalis*, *Bifidobacterium breve*, *Gardnerella vaginalis*, *Oxalobacter formigenes*, *Pediococcus pentosaceus*, *Enterococcus faecium*, *Collinsella intestinalis*, *Desulfovibrio piger*, *Lactobacillus reuteri*, *Peptostreptococcus anaerobius*, *Mitsuokella multacida*, *Eggerthella lenta*, *Bifidobacterium animalis*, *Mollicutes bacterium*, *Turicibacter* sp., *Bifidobacterium bifidum*, *Streptococcus mitis*, *Fusobacterium mortiferum*, *Lactococcus lactis*, *Streptococcus parasanguinis*, *Acidaminococcus fermentans*, *Klebsiella pneumoniae*, *Megamonas hypermegale*, *Lactobacillus ruminis*, *Streptococcus thermophilus*, *Clostridium asparagiforme*, *Bifidobacterium pseudocatenulatum*, *Clostridium bartlettii*, *Blautia hydrogenotrophica*, *Clostridium spiroforme*, *Catenibacterium mitsuokai*, *Clostridiales bacterium*, *Ruminococcus* sp., *Methanobrevibacter smithii*, *Veillonella parvula*, *Clostridium hathewayi*, *Eubacterium biforme*, *Clostridium bolteae*, *Holdemania filiformis*, *Bifidobacterium longum*, *Porphyromonas uenonis*, *Blautia hansenii*, *Collinsella aerofaciens*, *Erysipelotrichaceae bacterium*, *Eubacterium dolichum*, *Bifidobacterium adolescentis*, *Acidaminococcus* sp., *Eubacterium cylindroides*, *Clostridium leptum*, *Eubacterium hallii*, *Ruminococcus obeum*, *Anaerotruncus colihominis*, *Butyrate-prod. bacterium*, *Prevotella timonensis*, *Coprococcus catus*, *Ruminococcus gnavus*, *Clostridium nexile*, *Ruminococcus obeum*, *Clostridium* sp., *Ruminococcus lactaris*, *Coprococcus eutactus*, *Dorea formicigenerans*, *Dorea longicatena*, *Eubacterium ventriosum*, *Bacteroides pectinophilus*, *Ruminococcus* sp., *Coprococcus comes*, *Clostridium* sp., *Akkermansia muciniphila*, *Ruminococcus torques*, *Ruminococcus torques*, *Roseburia intestinalis*, *Eubacterium siraeum*, *Eubacterium eligens*, *Roseburia inulinivorans*, *Faecalibacterium prausnitzii*, *Escherichia coli*, *Butyrivibrio crossotus*, *Dialister invisus*, *Bacteroides cellulosilyticus*, *Ruminococcus bromii*, *Bacteroides coprophilus*, *Bacteroides eggerthii*, *Alistipes shahii*, *Eubacterium rectale*, *Parabacteroides* sp., *Bacteroides* sp., *Bacteroides plebeius*, *Bacteroides coprocola*, *Parabacteroides merdae*, *Prevotella copri*, *Alistipes putredinis*, *Bacteroides uniformis*, *Bacteroides ovatus*, *Bacteroides vulgatus.*	66 species

**Table 2:** Current methods for studying the intestinal microbiome.

Technique	DNA Sequencing Techniques		RNA Sequencing Techniques	Metabolomic Techniques	Metaproteomic Techniques
Methods	16S rRNA Gene Sequencing	WGS Sequencing	Meta-Transcriptomics		
Role	Identifies and classifies bacterial species within the microbiome based on their 16S rRNA genes	Characterization of the genetic and genomic diversity and functional potential of the microbial domains; assign taxonomy at the species and strain levels	Capture real-time responses to environmental changes; potential to uncover biological information	Study the functional potential of the microbiota by analyzing the metabolites produced by the microbiome	Provides information on protein expression and function and allows the investigation of host-microbe interactions; less sensitive than DNA/RNA-based methods
Advantages	Allow the analysis of multiple samples simultaneously Relatively inexpensive compared to whole-genome sequencing	It offers the possibility to assign taxonomy at the species and strain levels Comprehensive genomic information	Offer functional insight into microbial activity Broad taxonomic and functional coverage (provide information on all types of organisms within a sample) Offer the possibility to identify active metabolic pathways and study host-microbiome interactions	Functional insights Biomarker discovery Offer the possibility to discover metabolic pathways	Direct measurement of proteins Comprehensive functional profiling
Disadvantages	Ineffective for determining non-bacterial organisms Limited taxonomic resolution Unable to measure functional potential	Relatively expensive Difficult to analyze and interpret data	Increased costs Instability of RNA samples	Increased costs Difficult to analyze and interpret data Limited functional interpretation	Increased costs Difficulty in extracting and stabilizing protein samples

## Data Availability

Not applicable.
